# Advancing Precision Medicine for the Diagnosis and Treatment of Acute Respiratory Distress Syndrome

**DOI:** 10.3390/jcm12041563

**Published:** 2023-02-16

**Authors:** Alicia N. Rizzo, Neil R. Aggarwal, B. Taylor Thompson, Eric P. Schmidt

**Affiliations:** 1 Division of Pulmonary and Critical Care Medicine, Massachusetts General Hospital, Harvard Medical School, Boston, MA 02144, USA; 2 Division of Pulmonary Sciences and Critical Care Medicine, University of Colorado School of Medicine, Aurora, CO 80045, USA

**Keywords:** acute respiratory distress syndrome, heterogeneity, endotyping, precision medicine, mechanical ventilation

## Abstract

Acute respiratory distress syndrome (ARDS) is a common and life-threatening cause of respiratory failure. Despite decades of research, there are no effective pharmacologic therapies to treat this disease process and mortality remains high. The shortcomings of prior translational research efforts have been increasingly attributed to the heterogeneity of this complex syndrome, which has led to an increased focus on elucidating the mechanisms underlying the interpersonal heterogeneity of ARDS. This shift in focus aims to move the field towards personalized medicine by defining subgroups of ARDS patients with distinct biology, termed endotypes, to quickly identify patients that are most likely to benefit from mechanism targeted treatments. In this review, we first provide a historical perspective and review the key clinical trials that have advanced ARDS treatment. We then review the key challenges that exist with regards to the identification of treatable traits and the implementation of personalized medicine approaches in ARDS. Lastly, we discuss potential strategies and recommendations for future research that we believe will aid in both understanding the molecular pathogenesis of ARDS and the development of personalized treatment approaches.

## 1. Introduction and Historical Perspective

Acute Respiratory Distress Syndrome (ARDS) is a common disease process that affects over 200,000 U.S. patients per year, representing approximately one quarter of all mechanically ventilated patients, and is associated with substantial morbidity and a mortality rate of 30–40% [1,2]. Despite decades of research, there are still relatively few effective treatments for this disease and supportive management aimed at minimizing ventilator-induced lung injury remains the mainstay of treatment [3]. Although numerous potential pharmacologic agents have shown promise in animal models, and small clinical trials in ARDS, large randomized controlled trials (RCTs) of these pharmacologic agents have been largely disappointing [4]. This disconnect is increasingly attributed to the patient-level heterogeneity of this complex syndrome, which has led to a shift in the field towards understanding the mechanistic heterogeneity of ARDS so that potential therapeutic agents can be employed in the specific subgroups of patients that are most likely to derive benefit [5,6].

Interestingly, heterogeneity has been noted in ARDS since its original description in 1967, in which Ashbaugh and colleagues described a case series of 12 patients with acute hypoxemic respiratory failure due to several distinct inflammatory stimuli [7]. Given the lack of one dominant pathophysiologic mechanism to define this complex disease process, the definition has remained purely clinical through multiple revisions to the diagnostic criterion [8,9]. The current Berlin definition dictates that the precipitating illness must have occurred within one week of presentation, that patients must have bilateral infiltrates on imaging, and that the presentation cannot be solely explained by cardiogenic pulmonary edema [9]. The severity of the illness is then defined as mild, moderate, or severe based on the degree of hypoxemia as measured by PaO_2_:FiO_2_ while receiving a minimum of 5 cm H_2_O of positive end-expiratory pressure (PEEP) [9]. At autopsy, ARDS is classically characterized by diffuse alveolar damage (DAD), which describes an injury pattern that includes destruction of alveolar structure, the presence of hyaline membranes, infiltration of leukocytes, and the deposition of fibrin; however, more recent studies indicate that DAD is present in post-mortem evaluation in approximately 45% of patients who meet the current clinical definition of ARDS and was a more common finding in the high tidal volume era [10,11]. Pneumonia without hyaline membranes or DAD was the second most common histologic finding. These data underscore the heterogeneity underlying this disease processes and highlight an urgent need for improved noninvasive diagnostic testing to advance our approach to ARDS.

The first clinical trial to demonstrate a mortality benefit in ARDS was the ARMA trial, which demonstrated an 8.8% decrease in mortality (31% vs. 39.8%) in patients treated with lower tidal volume ventilation (6 mL/kg vs. 12 mL/kg of ideal body weight (IBW)) [3]. This groundbreaking work paved the way for other clinical trials that have helped us fine tune our approach to the supportive management of ARDS patients. This work included the FACTT trial, which demonstrated an increase in ventilator-free days in patients treated with a conservative fluid management approach and the PROSEVA trial which demonstrated decreased mortality with prone positioning in ARDS patients with a PaO_2_:FiO_2_ less than 150 [12,13]. By selectively enrolling patients with increased disease severity (PaO_2_:FiO_2_ < 150), the PROSEVA trial employed a strategy of prognostic enrichment, which describes selection of patients with a higher likelihood of experiencing the primary study outcome [5]. This strategy, which increases the likelihood of detecting a difference in the outcome of interest with a lower sample size for a given effect size, if one is present, has also been utilized in ARDS trials examining the efficacy of other supportive interventions in ARDS, such as neuromuscular blockade [14,15].

In contrast to the consistent improvement in outcomes achieved by optimizing supportive care for ARDS patients, clinical trials of pharmacologic therapies for ARDS have been largely disappointing, despite promising preclinical data [4,16,17,18,19]. The results of this collective literature suggest that further attempts to apply the same pharmacologic agent to all ARDS patients are unlikely to be fruitful and that future of ARDS care will require a precision medicine approach. Precision medicine, which is defined as matching therapies to individuals or subgroups of patients most likely to derive benefit, has revolutionized the care of many chronic disease processes, including asthma and many forms of cancer [5,20]. However, the development of precision medicine approaches for ARDS requires both understanding of the mechanistic heterogeneity of this complex syndrome and methods to predict treatment response at an individual patient’s bedside. Thus, although prognostic enrichment strategies have identified effective ways to improve the supportive management of ARDS, more sophisticated approaches will be necessary in order to develop effective pharmacologic therapies. Specifically, clinical trials involving strategies such as predictive enrichment, which entails enrolling patients who are most likely to respond to a particular treatment based on pathobiological characteristics, such as elevated levels of a biomarker that is suggestive of a particular dominant pathophysiologic mechanism, are needed [5,21]. Selected enrichment strategies used or proposed for ARDS clinical trials were recently reviewed in the ARJCMB (Table 1). In this section, we will discuss four unique challenges that the critical care research community faces with regards to the implementation of precision medicine approaches in critical illness syndromes such as ARDS.

## 2. Challenges in the Implementation of Precision Medicine in ARDS

### 2.1. Heterogeneity of the Molecular Pathogenesis of Injury

It is well established that the pathogenesis of ARDS involves multiple distinct, but related, mechanisms of injury including epithelial injury, endothelial injury, alveolar–capillary barrier dysfunction, impaired alveolar fluid clearance, surfactant dysfunction, and dysregulation of both inflammation and coagulation [36]. The cellular and molecular mechanisms that underlie each of these components of ARDS pathogenesis have been the topic of extensive laboratory science investigation using cell culture and small animal models of disease. Although these models are highly valuable to elucidate individual cellular signaling pathways, they are unable to fully recapitulate the complexity and heterogeneity of human ARDS stemming from diversity in the timing of onset and relative severity of the underlying ARDS risk factor, as well as patient-level factors including co-morbid conditions and genetic factors. As such, large observational cohort studies are necessary to discern the specific injury mechanisms that are responsible for ARDS heterogeneity and to identify the extent to which a given type of lung injury is driving ARDS pathogenesis in an individual patient.

As the importance of ARDS heterogeneity became increasingly appreciated, initial attempts to subgroup patients by mechanism of injury were based on the hypothesis that “direct lung injury” stemming from lung-focused insults such as pneumonia and aspiration would be characterized by more prominent epithelial injury, and “indirect lung injury” stemming from systemic insults such as sepsis and pancreatitis would be characterized by more endothelial lung injury. Indeed, multiple studies have confirmed that epithelial lung injury biomarkers, such as the receptor for the advanced glycation end products (RAGE) and surfactant protein D (SP-D), are elevated in patients with clinically-adjudicated direct lung injury and endothelial lung injury biomarkers, such as angiopoietin 2 (Ang-2), are elevated in patients thought to have indirect lung injury [37,38,39,40,41,42]. Although helpful, an approach based on the primary route and location of insult is likely an oversimplification of the complexity of ARDS heterogeneity, given the frequency at which ICU patients are simultaneously exposed to multiple lung injurious insults [41]. For example, a patient admitted with a lobar pneumonia who subsequently develops septic shock and ARDS may have elements of both direct injury from the pneumonia and indirect injury from the systemic inflammatory response and septic shock. Additionally, multiple aspects of the supportive management of these conditions, including alveolar overdistension from mechanical ventilation and endothelial injury from overly aggressive fluid resuscitation may also impact these injury patterns [43,44]. To this end, variability in the application of these interventions to ARDS patients in clinical practice is also important to consider in the design of clinical trials [45].

### 2.2. Limited Clinical Availability of ARDS Biomarkers

Although biomarkers of epithelial and endothelial injury (RAGE and Ang-2, respectively), vascular leak (BAL albumin), and the innate inflammatory response (IL-6, IL-8, and tumor necrosis factor alpha) [38,46] have been identified in small animal models and patients with ARDS, there are multiple barriers that currently limit the utility of these biomarkers in clinical practice. Specifically, most of the potential ARDS biomarkers identified in pre-clinical studies are not typically measured in routine clinical practice, or even in ARDS clinical trials, which severely limits our ability to retrospectively evaluate whether these biomarkers can predict outcomes or treatment responsiveness [38]. Additionally, in contrast to the murine pre-clinical data, which relies heavily on analysis of bronchoalveolar lavage (BAL) samples, prior human ARDS research has predominantly collected plasma, which does not necessarily reflect the inflammatory response in the lungs [47]. The relative rarity of airspace fluid samples from ARDS patients is likely driven by the lack of a proven, acceptable risk:benefit ratio of bronchoscopy and BAL for patents with ARDS [38]. Furthermore, given variability of fluid return during BAL there are also limitations to the interpretation of biomarker concentrations using this technique [48]. These limitations have led investigators to develop other methods of sampling the airspace fluid in ARDS patients [47,49]. Specifically, one novel method of obtaining airspace fluid noninvasively involves the use of heat moisture exchange (HME) filters, devices utilized to humidify the airways of ventilated patients as part of routine clinical practice in many ICUs [47]. Fluid collected from discarded HME filters closely recapitulated directly aspirated pulmonary edema fluid and thus can be used to measure ARDS biomarkers including RAGE, Ang-2, and protein [47,50,51]. Thus, large scale, multi-institutional efforts to collect HME filter fluid could provide valuable insights to the study of ARDS heterogeneity with little to no risk to patients.

### 2.3. Timing Issues: Rapid Illness Progression and Imprecise Disease Staging

An additional critical barrier to the implementation of precision medicine in ARDS care pertains to the rapidly progressive nature of this disease process. Notably, most disciplines that have successfully implemented precision-based medicine have applied these strategies to the treatment of chronic medical conditions. Chronic disease processes afford the clinician (and researcher) time to conduct sophisticated diagnostic tests, such as genetic testing to identify mutations that can predict responsiveness to specific chemotherapeutic agents [5]. In contrast, ARDS progresses rapidly, frequently over the course of hours to days, and patients are too acutely ill to undergo biopsies or other invasive diagnostic testing, and there is insufficient time to await test results. Thus, for diagnostic testing to provide substantial value in the bedside identification of ARDS pathogenesis, tests must be developed that are both rapid and minimally invasive. This critical point was highlighted during the ROSE trial, where 23% of all deaths occurred in the first 48 h [14]. Thus, diagnostics with the potential to impact management and early outcomes will need to be available in minutes to hours.

Additionally, the application of other precision-based medicine strategies, such as targeted cancer therapeutics, relies on precisely defined disease staging systems [36]. It is well described that ARDS progresses through an exudative phase, which is characterized by innate immune cell-mediated damage to endothelial and epithelial cells and the accumulation of edema fluid, a proliferative phase, in which repair processes are initiated in order to restore barrier integrity, and a final fibrotic phase, which occurs in a subset of ARDS patients who develop irreversible fibrosis [36]. Although existence of these stages is well established, the factors that drive progression through distinct ARDS stages are unknown and there are no clinically available tests to precisely determine what ARDS stage an individual patient is in, short of a lung biopsy with generously sized tissue samples. This is critical, as prior work has demonstrated that treatment responses in ARDS can differ based on stage of illness, at least using time from syndrome onset. For example, in the DEXA ARDS study, corticosteroid administration decreased mortality in patients who were treated early in their disease course; however, the LASRS study identified a signal towards harm when corticosteroids were administered after 14 days of unresolving ARDS [23,52]. Thus, potentially narrow, and time-sensitive therapeutic windows necessitate additional research to precisely define the stages of ARDS in order to optimize precision medicine approaches.

### 2.4. ARDS Heterogeneity as a Function of the Critical Care Environment

In addition to the above issues, it is critical to mention that the heterogeneity of ARDS pathophysiology and outcomes is also impacted by the heterogeneity of the treatments administered to these patients in our ICUs. For example, despite wide-spread acceptance of the results of the 2000 ARMA trial, which demonstrated decreased mortality with low tidal volume ventilation, the LUNG SAFE study, which was published in 2016, demonstrated that more than 33% of patients with ARDS received a tidal volume of >8 mL/kg of IBW [53]. There are multiple possible factors contributing to this unwarranted variability of care delivery, including delayed recognition of ARDS and overestimation of the necessary tidal volume in patients of short stature [53,54]. Efforts are underway to reduce this variability such as electronic surveillance systems to improve early recognition of ARDS; however, these technologies have not yet been incorporated into routine clinical practice [55].

In addition to the incomplete administration of proven therapies (such as low tidal volume ventilation), there is also significant variation in the application of many other aspects of ARDS management for which “optimum therapy” has yet to be proven. This includes aspects of ventilator management (e.g., selection of best PEEP), volume resuscitation practices, and the provision of extracorporeal membrane oxygenation (ECMO) [56,57,58]. Variations in clinical practice with regards to these, and other aspects of care, add additional heterogeneity to ARDS. For example, given that fluid resuscitation has been shown to contribute to endothelial injury, variation in resuscitation practices may contribute to the observed heterogeneity of both levels of endothelial injury markers and patient outcomes in ARDS [39,43]. Furthermore, variation in critical care resource availability also contributes to heterogeneity in care delivery to ARDS patients. The importance of this concept was highlighted during the COVID-19 pandemic, during which the geography and timing of massive surges in ARDS cases significantly impacted the ability of hospitals to administer care to COVID patients [59]. However, it is critical to note that variability in clinical resource availability is also present non-pandemic circumstances, which is particularly notable when comparing the ICUs in high-income countries to ICUs in lower- or middle-income countries [20].

## 3. Implementation Strategies

Although the complex issues described in the preceding section present a challenging task, recent work by basic, translational, and clinical researchers have led to new multi-disciplinary approaches to address these problems. In this section we will focus on four novel approaches to the study of ARDS heterogeneity that we believe have high likelihood of advancing the field of precision medicine in ARDS, as well as other complex critical illness syndromes.

### 3.1. Novel Approaches to ARDS Endotyping

In a landmark study, Calfee and colleagues utilized latent class analysis, a statistical model method that identifies unobserved groups within a heterogeneous population, to identify ARDS phenotypes based on the available clinical data from the participants in the ARMA and ALVEOLI trials [2,60]. This technique identified two ARDS phenotypes; a phenotype termed “hyperinflammatory”, which is defined by higher plasma concentrations of inflammatory cytokines, lower serum bicarbonate, and higher vasopressor requirements, and a phenotype termed “hypoinflammatory”, which is defined by lower concentrations of inflammatory cytokines, higher serum bicarbonate, and lower vasopressor requirements [2]. Importantly, these phenotypes were not segregated by illness severity scoring and have been subsequently validated in multiple large observational cohorts including the Validating Acute Lung Injury markers for Diagnosis (VALID) cohort at Vanderbilt and the Early Assessment of Renal and Lung Injury (EARLI) cohort at University of California, San Francisco [61].

Interestingly, post-hoc analysis of additional ARDS randomized controlled trials have demonstrated that these phenotypes have different responses to randomized treatments, results that were obscured in the original clinical trials that included all ARDS patients as a single group [33,62]. For example, patients with the hyperinflammatory phenotype experienced lower mortality with a higher PEEP strategy, liberal fluid management, and simvastatin, whereas patients with the hypoinflammatory phenotype either did not respond or experienced higher mortality when exposed to the same treatments [33,63,64,65]. Additionally, among COVID19 patients, the hyperinflammatory phenotype had an improved response to corticosteroids compared to the hypoinflammatory phenotype [62]. Together, this work demonstrates that subgroups of ARDS may have differential responses to therapy and underscores the importance and urgency of research to prospectively identify treatment responsive subgroups as well as biomarkers that can identify these subgroups at the bedside. A current limitation of this work is that there are not yet tests available to distinguish these phenotypes at the bedside as many of the markers that were used to derive these groups are not regularly tested in routine clinical practice.

### 3.2. Bedside-to-Bench Approaches for the Identification of Treatable Traits

While animal models are imperfect for the study of complex critical illnesses such as sepsis and ARDS, they remain crucial to the study of individual pathways that drive lung injury [5,66]. We advocate for the use of animal models within a bedside-to-bench approach that uses clinical data to identify pathways most likely to be fruitful for development of novel therapies that then can be tested in relevant animal models. In this research strategy, large observational trials of ARDS patients can be used to identify biomarkers that are associated with disease severity, clinical outcomes, or particular stages of disease. The findings of these exploratory analyses can then direct further mechanistic investigation into the pathways that are of the highest clinical significance. For example, our prior work demonstrated that degradation of the alveolar epithelial glycocalyx occurs in a subgroup of ARDS patients and that the degree of glycocalyx degradation is highly associated with the severity and duration of respiratory failure [51]. Additional analysis of the human sample data, as well as data from murine lung injury models, suggests that glycocalyx degradation occurs due to upregulation of matrix metalloproteinases (MMPs) that cleave proteoglycans from the epithelial surface, likely leading to impaired surfactant function [51,67,68,69]. This is critical, as surfactant dysfunction is a central aspect of ARDS pathophysiology and suggests that biomarkers of glycocalyx degradation may be used to identify patients in which surfactant dysfunction is a key driver of ARDS. These and other “bedside to bench” insights can then return to the bedside, as demonstrated by our development of a rapid point-of-care assay that can quantify glycocalyx shedding in biological specimens such as HME fluid and urine [51,70], potentially enabling the implementation of precision medicine approaches in the ICU.

### 3.3. Incorporating Anatomic and Physiologic Heterogeneity in ARDS Endotypes

In addition to the molecular heterogeneity of ARDS, there is also significant anatomic and physiologic heterogeneity that contributes to this complex syndrome. This includes factors that are patient-specific, such as obesity, and those that are injury-specific, such as the distribution of pulmonary opacities, both of which substantially change lung mechanics, and may impact the optimal mechanical ventilation strategy [71,72,73]. When ARDS was first described, chest radiography was the sole chest imaging modality available in most ICUs, which substantially limited our ability to study the distribution of lung injury in ARDS [7]. However, in the 1980s, as computed tomography (CT) scanners became more widely available, it was quickly noted that there is significant anteroposterior variability in lung injury, which led Gattinoni and colleagues to describe the concept of the “baby lung” in which the ARDS lung is effectively smaller due to the presence of dependent atelectasis and consolidations [74]. This work has enormous implications in terms of the management of ARDS as these findings helped form the basis for the benefit of low tidal volume ventilation and prone positioning [3,12].

Subsequently, additional work has led to the identification of radiographic subtypes of ARDS defined as nonfocal/diffuse and focal/lobar [74,75]. The biologic basis and critical importance of these phenotypes is supported by the observations that nonfocal/diffuse ARDS is more frequently due to systemic insults that indirectly induce lung injury, is associated with lower levels of RAGE (which reflects epithelial damage), and results in worse lung compliance and higher mortality [15,72,76]. Recognition of these distinct radiographic phenotypes led to the LIVE trials, which attempted to personalize mechanical ventilation strategy with the hypothesis that individuals with focal/lobar ARDS have an increased volume of normal lung parenchyma, and thus would tolerate higher tidal volumes than those with nonfocal/diffuse ARDS [26]. Although these trials did not demonstrate a mortality benefit, these results may have been impacted by misclassification of a significant number of patients in the study [26]. Additionally, further study in this area has demonstrated that patients with COVID-19 ARDS have a higher lung gas volume as measured on CT chest for a given PaO_2_:FiO_2_ and suggests that the optimum mechanical ventilation strategy may be different between these two groups [77,78].

### 3.4. Innovative Clinical Trial Design

The massive increase in the incidence of ARDS during the COVID-19 pandemic created an urgent need to evaluate many potential treatments for COVID-19 disease including therapies specifically targeting COVID-19 induced ARDS. This critical care research community swiftly responded to this need by implementing large platform trials, including I-SPY COVID, the ACTIV suite of trials, and RECOVERY which were sponsored by the US and the UK [79,80]. Additionally, the WHO sponsored trial consortium quickly pivoted its plans for the REMAP-CAP trial to focus on the evaluating the efficacy of the numerous agents that had been proposed as treatments for COVID-19 [81]. These trials collectively identified the beneficial effect of dexamethasone, baricitinib, and tocilizumab in COVID-19 ARDS and discarded many therapies that were ineffective including hydroxychloroquine and ivermectin [6]. The efficiency of these trials is due to their platform trial design, in which multiple potential therapies are tested simultaneously against a single control group, which can dramatically improve trial efficiency as compared to a traditional randomized control trial that typically tests one therapy at a time [5]. Platform trials are frequently adaptive in nature, allowing for potential therapies to be added or removed from the protocol based on interim data analysis without completely interrupting trial enrollment. Additionally, these trials are typically designed to allow for updates to the standard of care treatment as successful treatments are identified, which is another key aspect to the efficiency of this method [82,83]. Although the complex nature of these trials requires an extremely organized and collaborative effort and highly sophisticated statistical analyses, this work serves as proof of concept that these types of trials are feasible in the ICU setting.

## 4. Conclusions

The immense clinical and pathophysiologic heterogeneity intrinsic to ARDS necessitates precision medicine approaches to both diagnosis and treatment. Although decades of prior randomized controlled trials of pharmacologic agents in ARDS have largely failed to identify agents that decrease mortality, these trials, with the foresight to collect biospecimens, and the mechanistic basic science literature prior to and concurrent with them, lay the foundation for a new era of ARDS research that focuses on the implementation of precision medicine. Over the past several years, investigators have identified multiple subgroups of ARDS that are defined by several heterogeneous aspects of this disease process including host factors (e.g., medical co-morbidities and genetic factors), etiology and timing of injury, radiographic injury patterns, and disease severity. Critically, some of these phenotypes have demonstrated differential responses to pharmacologic therapies when evaluated retrospectively, which revealed the important finding that the broad application of pharmacologic agents in ARDS, without concurrent endophenotyping, may both impair our ability to detect a positive signaling in certain phenotypes and mask our ability to detect potential harms in other subgroups.

Future work must validate biomarkers that can detect treatable traits within ARDS and develop panels of biomarkers, which will ideally include measures of both the pulmonary and systemic inflammatory responses, in order to identify the specific mechanisms that are driving injury in an individual patient. Given the rapidly progressive nature of ARDS, we believe that an emphasis should be placed on the development of rapid point-of-care assays for these biomarkers to utilize them prospectively in clinical studies for predictive enrichment. Additionally, more work is needed to understand the impact of anatomic and physiologic heterogeneity of ARDS due to both patient specific-factors (e.g., obesity) and disease-specific heterogeneity (e.g., radiographic distribution of lung injury) as these factors have important effects on lung mechanics and are thus likely to impact the optimum mechanical ventilation strategy. Lastly, this work will all need to be interpreted in the context of clinical practice variability for patients with ARDS. We believe that a concerted effort by the critical care community to study and address these issues will allow us to apply precision therapies for ARDS and ultimately improve outcomes for our patients.

## Figures and Tables

**Table 1:** Selected enrichment strategies used or proposed for ARDS clinical trials. Reprinted with permission of the American Thoracic Society. Copyright © 2022 American Thoracic Society. All rights reserved. Cite: Martin TR, Zemans RL, Ware LB, Schmidt EP, Riches DWH, Bastarache L, Calfee CS, Desai TJ, Herold S, Hough CL, Looney MR, Matthay MA, Meyer N, Parikh SM, Stevens T, Thompson BT. New Insights into Clinical and Mechanistic Heterogeneity of the Acute Respiratory Distress Syndrome: Summary of the Aspen Lung Conference 2021. Am J Respir Cell Mol Biol. 2022 Sep;67(3):284–308. Doi: 10.1165/rcmb.2022-0089WS. PMID: 35679511; PMCID: PMC9447141. The American Journal of Respiratory Cell and Molecular Biology is an official journal of the American Thoracic Society.

Trial/Author	Enrichment Strategy	Intervention	Findings/Rationale
ACURASYS, Papazian [22] ROSE, PETAL Network [14]	ARDS Severity PF < 120–150 P/F < 120	Early neuromuscular blockade	ACURASYS demonstrated higher placebo mortality in, and benefits limited to, the P/F < 120 subsets (prognostic and predictive enrichment, respectively). Did not replicate in ROSE.
PROSEVA, Guerin [12]	ARDS Severity P/F < 150	Prone positioning	Large treatment effect in moderate to severe ARDS concordant with prior metanalyses suggesting predictive enrichment.
LASRS, Steinberg [23]	ARDS for 7–28 d	Methylprednisolone	Attempted to enrich for a steroid-responsive phase of ARDS (fibro-proliferation). Late steroids (>14 d) may be harmful.
Willson [24] Spragg [25]	Direct vs. indirect	Surfactant replacement	Benefit with pediatric direct lung injury. Did not replicate in adults.
Constantin [26]	Focal vs. diffuse ARDS	Personalized ventilator strategy; higher V_T_ and lower PEEP for focal vs. lower V_T_ and higher PEEP for diffuse ARDS	No difference in mortality; high rates of misclassification and higher mortality if a strategy is applied to the incorrect subgroup.
Calfee [27]	Trauma vs non-trauma	Reduce heterogeneity by studying traumatic ARDS separately	Lower mortality is not explained by baseline clinical factors; biomarker profiles suggest the differing extent of epithelial and endothelial injury.
Villar [28] Goligher [29]	Evaluate stability on standardized ventilator settings Assess physiologic responsiveness during a run-in period	Enroll only persistent ARDS Randomize to higher vs. lower PEEP in responders only	Reevaluation after 24 h enriches for higher mortality. Analysis of PEEP responsiveness in RCTs suggests a potential for predictive and prognostic enrichment.
Gattinoni [30] Goligher [31,32]	Match lung-protective intervention to physiology to optimize benefit/risk	Assess for recruitability or lung weight (CT) ECCO2R for subset likely to have a ≥5 cm H_2_O drop in driving pressure Titration of tidal volume to elastance	Modeling and observational data suggest potential for both prognostic and predictive enrichment.
Calfee [33]	ARDS subclass	Simvastatin for Class 2 (“Hyperinflammatory”) ARDS (see text)	Post hoc analysis of RCT demonstrates mortality benefit limited to Class 2 ARDS.
Lai [34] Sinha [35]	Markers of dysregulated coagulation, high dead space fraction or ventilatory ratio, and RV function by cardiac ultrasound	Anticoagulants or pulmonary vascular targeted therapies	Identify subsets with or at risk for microvascular thrombi, vascular remodeling, pulmonary hypertension, or adverse outcomes.

Definition of abbreviations: ACURASYS = ARDS et Curarisation Systematique; ARDS = acute respiratory distress syndrome; ECCO_2_R = extracorporeal CO_2_ Removal; LASRS = Late Steroid Rescue Study; PEEP = positive end-expiratory pressure; PETAL = Prevention and Early Treatment of Acute Lung Injury; PROSEVA = Proning Severe ARDS Patients; RCT = Randomized Clinical Trial; ROSE = Reevaluation of Systemic Early Neuromuscular Blockade; V_T_ = tidal volume.
